# Sodium Consumption: An Individual's Choice?

**DOI:** 10.1155/2012/860954

**Published:** 2012-01-03

**Authors:** Norm R. C. Campbell, Jillian A. Johnson, Tavis S. Campbell

**Affiliations:** ^1^Departments of Medicine, of Community Health Sciences, and of Physiology and Pharmacology, Libin Cardiovascular Institute, University of Calgary, 3280 Hospital Drive NW, Calgary, AB, Canada T2N 4Z6; ^2^Department of Psychology, University of Calgary, 2500 University Drive NW, Calgary, AB, Canada T2N 1N4

## Abstract

Excess intake of dietary salt is estimated to be one of the leading risks to health worldwide. Major national and international health organizations, along with many governments around the world, have called for reductions in the consumption of dietary salt. This paper discusses behavioural and population interventions as mechanisms to reduce dietary salt. In developed countries, salt added during food processing is the dominant source of salt and largely outside of the direct control of individuals. Population-based interventions have the potential to improve health and to be cost saving for these countries. In developing economies, where salt added in cooking and at the table is the dominant source, interventions based on education and behaviour change have been estimated to be highly cost effective. Regardless, countries with either developed or developing economies can benefit from the integration of both population and behavioural change interventions.

## 1. Introduction

Cardiovascular diseases are the leading cause of death worldwide [1]. In 2008, an estimated 17.3 million people died from cardiovascular disease [1]. Of those deaths, an estimated 7.3 million were due to coronary heart disease and 6.2 million due to stroke [1]. A disproportionate amount of those deaths, over 80%, take place in low- and middle-income countries and occur at similar rates among men and women [1]. Not surprisingly, elevated blood pressure levels are a major cause of these diseases and are found at higher rates among low- and middle-income countries [1]. The relationship between blood pressure levels and risk of developing cardiovascular disease is strong and well supported [2]. In 2008, approximately one billion adults worldwide had uncontrolled hypertension (defined as systolic blood pressure ≥140 mm Hg systolic and/or diastolic blood pressure ≥90 mm Hg) [1]. Given the increasing prevalence of hypertension worldwide and the associated risk for developing cardiovascular disease, public health interventions aimed at reducing blood pressure are crucial. 

Many national and international agencies have acknowledged the role of lifestyle and diet, in particular sodium intake, on blood pressure levels. Diets high in salt are now recognized as one of the leading risks to cardiovascular health in the world as they increase blood pressure in both children and adults [3]. Furthermore, a recent meta-analysis of randomized trials has demonstrated that modest reductions in dietary sodium intake are associated with significant reductions in blood pressure in both normotensives and hypertensives and a 20% reduction in cardiovascular events [4, 5]. A reduction in salt intake of 6 g/day lowered blood pressure by 7/4 mm Hg diastolic in hypertensives and 4/2 mm Hg in normotensives [4]. This relationship has been empirically supported and is sufficiently strong to warrant recommendations for public health interventions aimed at substantially reducing dietary sodium intake. Furthermore, sodium reduction is noted as one of the most cost effective and most easily implemented strategies to improve population health [5–13]. Reducing dietary salt is recommended by the World Health Organization and many national governmental and nongovernmental health organizations. Some agencies, however, do not promote a reduction in dietary sodium, namely, nongovernmental or commercial organizations such as the Salt Institute, as they are sponsored by either the food or salt industries [9, 11, 14–17]. Regardless, it is apparent that the risks associated with an increase in salt consumption, chiefly those related to an increase in blood pressure, are linear [3, 18]. Most health economic models input relatively small changes in blood pressure that occur in those with normal and high blood pressure as estimated by short-term modest reductions in dietary sodium [7, 8, 19]. Some models also include the gastric cancers that are positively associated with, and probably caused by, high-salt intake [13]. Typically, the health economic models do not include the potential impact of longer term irreversible increases in blood pressure, age-related increases in blood pressure, the epigenetic phenomena, whereby exposure to excess salt in utero may increase blood pressure in offspring, or that sodium may increase vascular and cardiac disease in the absence of changes in blood pressure [7, 8, 12, 19–21]. The burden of disease studies also do not account for diseases that have a pathophysiological basis and close association with high sodium diets (i.e., increased severity and frequency of asthma attacks [22], increased calcium containing kidney stones [23], osteoporosis [23], or obesity related to the consumption of calorie containing beverages caused by sodium-induced thirst [24]). Hence the burden of disease associated with excess dietary salt is not only high, but may also be underestimated [20].

The objective of this commentary is to review current sodium consumption worldwide, discuss cost-effective strategies to reduce dietary sodium, as well as briefly review the role of behavioural and policy-based environmental interventions in reducing dietary sodium on a population-based scale.

## 2. Salt Consumption

Humans evolved on diets consisting of natural plant and animal foods containing small amounts of sodium, typically less than 2 g/day [25, 26]. Today, nearly all populations consume far greater quantities of salt than those provided in natural, unprocessed food diets. The World Health Organization currently recommends a daily consumption of less than 5 grams of salt [9], although some agencies recommend that no more than 1500 mg of sodium should be consumed per day [27–29], calculated as 2/3 tsp of table salt. In most populations, sodium intake is 5.7 g or more/day after age 5, with many populations consuming and average of over 10 g/day [30–32]. Furthermore, within high sodium consumption countries, only a small proportion of individuals consume the recommended levels of salt. For example, in Canada, a country with average salt consumption of 8.5 gm/day, 85% of men and 60% of women aged 9 to 70 consume over the upper recommended limit for salt and the vast majority (>90%) are above the level recommended for individuals to consume [33, 34].

Excess sodium intake results in adverse effects beyond those of increasing blood pressure. For example, one study found that in a population of overweight adults, a daily intake of sodium greater than 2300 mg/day was associated with a 61% increase in coronary heart disease mortality, an 89% increase in stroke mortality, and a 39% increase in all-cause mortality over a 19-year period [35]. Along with the other sodium-related illnesses discussed above (i.e., gastric cancers, kidney stones, etc.), it is clear that the economic costs associated with such illnesses can be substantial.

## 3. Cost Effectiveness of Interventions to Reduce Dietary Salt

In countries with developed economies, salt added during the processing of foods accounts for the vast majority of dietary salt (75–80%) [36]. An additional 10% of dietary salt is accounted for by salt that is naturally occurring in foods, while the rest is accounted for by salt added at the table or during cooking [36]. In low- to-middle income countries where populations may have limited access to processed foods, salt added at home, in cooking, or at the table, accounts for the majority of dietary salt [14]. Reducing dietary salt is estimated to save substantial health care costs [7, 10, 16, 30, 37–41]. For example, reducing dietary salt by 3 g/day in the United States is estimated to save 194,000 to 392,000 quality adjusted life years and reduce health care costs $10 to $24 billion US dollars a year [7]. In Canada, reducing salt consumption to recommended levels is estimated to reduce the prevalence of hypertension by 30% and to save up to $430 million dollars per year just in direct hypertension management costs alone [38]. In lower-income countries, programs aimed at reducing consumption of dietary salt through an intervention largely based on education are estimated to cost little (less than $0.40 USD per person per year), reduce premature deaths by close to 14 million in 10 years, and to be slightly more cost effective than strategies to reduce tobacco use (both highly advocated interventions) [8, 40].

## 4. Awareness and Barriers to Change

Although there is a general lack of awareness of salt as a health issue in many countries, some countries with established salt reduction programs show increasing awareness [41]. For example, in Canada, 80% of people diagnosed with hypertension are attempting to reduce dietary sodium [42]. In addition, many food companies have developed low-salt options to their product lines for people to choose, with some companies reducing salt additives in their full product line [43]. In developed economies, there are substantial barriers to free choice in those who chose to eat less salt [16, 44]. In most countries, nutritional information is not readily available. It is often the case that nutritional information is available only on the company's website, by asking for and reviewing a binder on site, or only readily available after purchase. Even in the United States and Canada, countries with mandatory labelling of packaged foods, the labels are often difficult to interpret. Also serving sizes may be variable and not comparable between products. In an unpublished study, we found that in a sample of over 100 people with diabetes who had received training on how to read a food label, not one could accurately answer how much of a processed food they could eat in a day when presented with the food label. In other countries, food labelling on packaged foods may not be mandatory and labelled foods may not be available. In remote regions and areas where populations vulnerable to the development of elevated blood pressure reside, low-salt alternative choices are typically not available. Furthermore, these populations may not have the health literacy with which to make informed choices. Food processors and manufacturers often use pervasive marketing techniques to create consumer demand for high-salt foods, which undermine efforts of public health and individual education interventions that attempt to reduce sodium intake. Moreover, these marketing techniques are often directed towards children by making consumption of such foods seem “fun” [45].

Perhaps the greatest barrier to choosing and maintaining a low-sodium diet is that high-salt foods are ubiquitous and hence difficult for those who choose low-salt diets [16, 46]. In Canada, high-salt diets are by and large perceived as unhealthy by the general population [47]. However, it is often the case that the same people who recognize that Canadians in general consume too much salt, believe that their personal consumption of dietary sodium is within the recommended amount [47]. This suggests that even a relatively affluent, well-educated population may have difficulty identifying and avoiding high-salt foods even if they perceive it is a health issue and have chosen to follow a low-salt diet. Some of the challenges of individual choice in selecting low salt diets is perhaps best illustrated by clinical trials where highly motivated patients are carefully and repeatedly trained how to select low-salt foods but generally can only sustain small reductions in salt intake long term [44, 48].

## 5. Population Interventions to Reduce Dietary Salt

Population-based approaches to reducing dietary salt may be effective in developed economies and have shown promising results for reducing blood pressure. In the late 1950s, the Japanese Government implemented a campaign to reduce salt intake given the high stroke mortality rates. Ten years later, salt intake was reduced from an average of 13.5 to 12.1 g/day overall, and from 18 to 14 g/day in the northern regions [49]. The reduction resulted in a decrease in average blood pressure and an 80% reduction in stroke mortality [49]. In the 1970s, Finland's government began a public education campaign and enforced regulations on food processing companies through a warning label on high-salt foods in order to reduce salt consumption across the country [50, 51]. More than 30 years later, the overall sodium intake in Finland has decreased more than 40%, with a subsequent decrease in mean diastolic blood pressure of greater than 10 mm Hg and an 80% decline in the mortality rate from heart disease and stroke [52]. Similar results were also seen on smaller scales in the DASH (Dietary Approached to Stop Hypertension) trial conducted in the USA [3, 53]. This trial assessed three levels of dietary sodium intake on two diets (American diet versus DASH diet) and demonstrated that reducing sodium in either diet resulted in lower blood pressure [3, 12, 53].

More recently in the United Kingdom, reductions in the amount of salt added to foods, in conjunction with a social marketing campaign, have been associated with reduction in population salt intake [14]. In developing economies widespread replacement of salt with a partial salt replacement (sodium, potassium magnesium combination) holds great promise [54, 55]. For example, a recent double-blind randomized controlled trial conducted in rural northern China found that replacing household salt with a reduced-sodium, high-potassium salt substitute for 1 year reduced systolic blood pressure by 5.4 mm Hg [55]. This low cost change in diet has shown promising outcomes for blood pressure reduction with little to no burden on the consumer.

## 6. Individual- and Population-Based Approaches

Clearly both a mix of population approaches and behavioural approaches targeting individuals are required to reduce sodium intake to within recommended levels [10, 11, 15, 16, 33, 56]. Similar methods have been successfully employed in reducing tobacco use [57]. With respect to individually targeted efforts, for example, a variety of behavioural interventions, including brief physician smoking cessation counselling, was found to meaningfully increase smoking quit rates [58]. However, it has become increasingly clear that education targeted towards individuals may be necessary, but not sufficient, to motivate long-term health behaviour change [59, 60]. Behavioural medicine researchers have begun testing and implementing more sophisticated models of behaviour change. For example, Motivational Interviewing, a directive patient-centred counselling approach focused on exploring and resolving ambivalence, which emerged as an effective therapeutic approach within the addictions field [61], has recently shown promise for other complex behaviour change problems such as weight loss in overweight and obese patients [62] and adherence to antihypertensive medication [63]. In contrast with recommendations for behaviour change delivered through education and advice giving, Motivational Interviewing differs in that motivation for change is elicited from individuals, rather than imparted by a healthcare provider [64].

The mix of behavioural interventions and population interventions depends on the specific circumstances of both the individual and the population. In countries with developed economies, population-based approaches, and a reduction of salt additives to food, supplemented by public education campaigns, need to be the primary means of intervention to ensure that the healthy option that is low in salt is the easiest option—a basic caveat of public health interventions. A universal reduction in salt additives during the manufacturing process has a strong potential to reduce health disparities in vulnerable populations while improving overall population health. Behavioural interventions may be most important to ensure the population and especially policy makers understand and are supportive of the need to reduce dietary salt. However, for specific individuals with strong motivation or at a greater personal risk from consuming a diet high in sodium, intensive behavioural interventions may be efficacious. Notably sole reliance on the individual behavioural approach is likely to have a smaller impact on a population basis, to be expensive, and to increase health disparity.

In developing economies, where the majority of sodium intake comes from salt added at the table and in cooking, behavioural interventions are more likely to be effective in reducing overall intake than population-based means [8, 32]. In this case, the individual needs to understand the consequences of excess sodium intake and change their behaviors (i.e., eating habits) to reduce sodium intake. Nevertheless, even in this setting, population interventions may still play an important role. Partial replacement of table salt (sodium chloride) with various mineral salts (mixtures of sodium, potassium, and/or magnesium and calcium) has been shown to be highly effective to reduce overall sodium consumption and also reduce blood pressure [55, 65]. Efforts to reduce dietary salt are also likely to reduce dietary iodine, therefore monitoring dietary iodine adequacy and revising the iodine content of salt is essential to maintain population health in most settings, including developed countries [66]. Population interventions that ensure widespread replacement of salt with a partial salt substitute that contains iodine may be the dominant strategy to reduce sodium intake on a large-scale basis in combination with behaviour change interventions.

## 7. Conclusion

Given the promising outcomes observed in recent randomized controlled trials and population-based interventions, reducing dietary sodium intake to modest levels (approximately 5 g/day) worldwide would result in a major improvement in overall health and reduce the costs associated with diseases connected to excess sodium intake. However, it is apparent that relying solely on interventions that target individual behaviour is not the ideal approach for reducing sodium consumption. While it may contribute to behaviour change among highly motivated individuals and increase the acceptability of population-based interventions, the latter approach seems better suited for this particular health behaviour.

## References

[1] World Health Organization Global status report on non-communicable diseases 2010.

[2] World Health Organization Prevention of cardiovascular disease: Guidelines for assessment and management of cardiovascular risk.

[3] Sacks FM, Svetkey LP, Vollmer WM (2001). Effects on blood pressure of reduced dietary sodium and the dietary approaches to stop hypertension (DASH) diet. *The New England Journal of Medicine*.

[4] He FJ, MacGregor GA (2002). Effect of modest salt reduction on blood pressure: a meta-analysis of randomized trials. Implications for public health. *Journal of Human Hypertension*.

[5] He FJ, MacGregor GA (2011). Salt reduction lowers cardiovascular risk: meta-analysis of outcome trials. *The Lancet*.

[6] He FJ, MacGregor GA (2011). Salt and cardiovascular disease mortality. *The Lancet*.

[7] Bibbins-Domingo K, Chertow GM, Coxson PG (2010). Projected effect of dietary salt reductions on future cardiovascular disease. *The New England Journal of Medicine*.

[8] Asaria P, Chisholm D, Mathers C, Ezzati M, Beaglehole R (2007). Chronic disease prevention: health effects and financial costs of strategies to reduce salt intake and control tobacco use. *The Lancet*.

[9] World Health Organization Creating an enabling environment for population-based salt reduction strategies.

[10] Campbell NRC, Legowski B, Legetic B (2011). Mobilising the Americas for dietary salt reduction. *The Lancet*.

[11] World Health Organization Reducing Salt Intake in Populations.

[12] He FJ, MacGregor GA (2009). A comprehensive review on salt and health and current experience of worldwide salt reduction programmes. *Journal of Human Hypertension*.

[13] Danaei G, Ding EL, Mozaffarian D (2009). The preventable causes of death in the United States: comparative risk assessment of dietary, lifestyle, and metabolic risk factors. *PLoS Medicine*.

[14] Webster JL, Dunford EK, Hawkes C, Neal BC (2011). Salt reduction initiatives around the world. *Journal of Hypertension*.

[15] World Health Organization Reducing Salt Intake in Populations.

[16] Henny JE, Taylor CL, Boon CS (2010). *Strategies to Reduce Sodium Intake in the United States*.

[17] Mohan S, Campbell NRC, Willis K (2009). Effective population-wide public health interventions to promote sodium reduction. *Canadian Medical Association Journal*.

[18] He FJ, MacGregor GA (2003). How far should salt intake be reduced?. *Hypertension*.

[19] Palar K, Sturm R (2009). Potential societal savings from reduced sodium consumption in the U.S. adult population. *American Journal of Health Promotion*.

[20] He FJ, MacGregor GA (2010). Reducing population salt intake worldwide: from evidence to implementation. *Progress in Cardiovascular Diseases*.

[21] van Vliet BN, Montani JP (2008). The time course of salt-induced hypertension, and why it matters. *International Journal of Obesity*.

[22] Mickleborough TD, Lindley MR, Ray S (2005). Dietary salt, airway inflammation, and diffusion capacity in exercise-induced asthma. *Medicine and Science in Sports and Exercise*.

[23] Cappuccio FP, Kalaitzidis R, Duneclift S, Eastwood JB (2000). Unravelling the links between calcium excretion, salt intake, hypertension, kidney stones and bone metabolism. *Journal of Nephrology*.

[24] He FJ, Marrero NM, MacGregor GA (2008). Salt intake is related to soft drink consumption in children and adolescents: a link to obesity?. *Hypertension*.

[25] Eaton SB, Konner M (1985). Paleolithic nutrition. A consideration of its nature and current implications. *The New England Journal of Medicine*.

[26] Eaton SB, Eaton SB (2000). Paleolithic vs. modern diets—selected pathophysiological implications. *European Journal of Nutrition*.

[27] Lloyd-Jones DM, Hong Y, Labarthe D (2010). Defining and setting national goals for cardiovascular health promotion and disease reduction: the american heart association’s strategic impact goal through 2020 and beyond. *Circulation*.

[28] Centers for Disease Control and Prevention (CDC) (2009). Application of lower sodium intake recommendations to adults—United States, 1999–2006. *Morbidity and Mortality Weekly Report*.

[29] Panel on Dietary Reference Intakes for Electrolytes and Water (2004). *Dietary Reference Intakes for Water, Potassium, Sodium, Chloride and Sulfate*.

[30] Brown IJ, Tzoulaki I, Candeias V, Elliott P (2009). Salt intakes around the world: implications for public health. *International Journal of Epidemiology*.

[31] Anderson CAM, Appel LJ, Okuda N (2010). Dietary sources of sodium in China, Japan, the United Kingdom, and the United States, women and men aged 40 to 59 years: the INTERMAP Study. *Journal of the American Dietetic Association*.

[32] Elliott P, Stamler J, Nichols R (1996). Intersalt revisited: further analyses of 24 hour sodium excretion and blood pressure within and across populations. *British Medical Journal*.

[33] Sodium Working Group (2010). *Sodium Reduction Strategy for Canada. Sodium Reduction*.

[34] Garriguet D (2007). Sodium consumption at all ages. *Health Reports*.

[35] He J, Ogden LG, Vupputuri S, Bazzano LA, Loria C, Whelton PK (1999). Dietary sodium intake and subsequent risk of cardiovascular disease in overweight adults. *Journal of the American Medical Association*.

[36] Mattes RD, Donnelly D (1991). Relative contributions of dietary sodium sources. *Journal of the American College of Nutrition*.

[37] Reboldi G, Gentile G, Angeli F, Verdecchia P (2010). Blood pressure lowering in the oldest old. *Journal of Hypertension*.

[38] Joffres MR, Campbell NRC, Manns B, Tu K (2007). Estimate of the benefits of a population-based reduction in dietary sodium additives on hypertension and its related health care costs in Canada. *Canadian Journal of Cardiology*.

[39] Wang G, Labarthe D (2011). The cost-effectiveness of interventions designed to reduce sodium intake. *Journal of Hypertension*.

[40] Beaglehole R, Bonita R, Horton R (2011). Priority actions for the non-communicable disease crisis. *The Lancet*.

[41] World Health Organization Strategies to monitor and evaluate population sodium consumptions and sources of sodium in the diet.

[42] Gee ME, Bienek A, Campbell NRC Prevalence of, and barriers to, preventative lifestyle behaviors in hypertension (from a national survey of Canadians with hypertension).

[43] World Health Organization Reducing salt intake in populations.

[44] Cobiac LJ, Vos T, Veerman JL (2010). Cost-effectiveness of interventions to reduce dietary salt intake. *Heart*.

[45] Elliott C (2008). Taste rules! food marketing, food law, and childhood obesity in Canada. *Journal of Canadian Food Cultures*.

[46] Fischer PWF, Vigneault M, Huang R, Arvaniti K, Roach P (2009). Sodium food sources in the Canadian diet. *Applied Physiology, Nutrition and Metabolism*.

[47] Decima Research (2009). Canadians’ and health care professionals’ views on sodium. *Final Report*.

[48] Hooper L, Bartlett C, Smith GD, Ebrahim S (2002). Systematic review of long term effects of advice to reduce dietary salt in adults. *British Medical Journal*.

[49] Sasaki N, Yamori Y (1979). The salt factor in apoplexy and hypertension: epidemiological studies in Japan. *Prophylactic Approach to Hypertensive Diseases*.

[50] Karppanen H, Mervaala E (2006). Sodium Intake and Hypertension. *Progress in Cardiovascular Diseases*.

[51] Pietinen P, Valsta LM, Hirvonen T, Sinkko H (2008). Labelling the salt content in foods: a useful tool in reducing sodium intake in Finland. *Public Health Nutrition*.

[52] Laatikainen T, Pietinen P, Valsta L, Sundvall J, Reinivuo H, Tuomilehto J (2006). Sodium in the Finnish diet: 20-year trends in urinary sodium excretion among the adult population. *European Journal of Clinical Nutrition*.

[53] Appel LJ, Brands MW, Daniels SR, Karanja N, Elmer PJ, Sacks FM (2006). Dietary approaches to prevent and treat hypertension: a scientific statement from the American Heart Association. *Hypertension*.

[54] He FJ, MacGregor GA (2008). Can a low-sodium, high-potassium salt substitute reduce blood pressure in rural Chinese people? Commentary. *Nature Clinical Practice Cardiovascular Medicine*.

[55] Li Y, Liu J, Song D (2007). Salt substitution: a low-cost strategy for blood pressure control among rural Chinese. A randomized, controlled trial. *Journal of Hypertension*.

[56] Scientific Advisory Committee on Nutrition (2003). *Salt and Health*.

[57] World Health Organization WHO Framework Convention on Tobacco Control.

[58] Okuyemi KS, Nollen NL, Ahluwalia JS (2006). Interventions to facilitate smoking cessation. *American Family Physician*.

[59] Bandura A, Schwarzer R (1992). Self-efficacy mechanism in psychobiologic functioning. *Self-Efficacy: Thought Control of Action*.

[60] Shumaker SA, Ockene JK, Riekert KA (2008). *The Handbook of Health Behavior Change*.

[61] Miller WR, Wilbourne PL (2002). Mesa grande: a methodological analysis of clinical trials of treatments for alcohol use disorders. *Addiction*.

[62] Armstrong MJ, Mottershead TA, Ronksley PE, Sigal RJ, Campbell TS, Hemmelgarn BR (2011). Motivational interviewing to improve weight loss in overweight and/or obese patients: a systematic review and meta-analysis of randomized controlled trials. *Obesity Reviews*.

[63] Ogedegbe G, Chapli W, Schoenthaler A (2008). A practive-based trial of motivational interviewing and adherence in hypertensive African Amercians. *American Journal of Hypertension*.

[64] Miller WR, Rollnich S (2002). *Motivational Interviewing: Preparing People for Change*.

[65] Mu J, Liu Z, Liu F, Xu X, Liang Y, Zhu D (2009). Family-based randomized trial to detect effects on blood pressure of a salt substitute containing potassium and calcium in hypertensive adolescents. *American Journal of Hypertension*.

[66] World Health Organization Salt as a Vehicle for Fortification.

